# Risk-reducing salpingectomy with delayed oophorectomy to prevent ovarian cancer in women with an increased inherited risk: insights into an alternative strategy

**DOI:** 10.1007/s10689-024-00412-0

**Published:** 2024-06-21

**Authors:** TA Gootzen, MP Steenbeek, MHD van Bommel, J IntHout, CM Kets, RPMG Hermens, JA de Hullu

**Affiliations:** 1 Department of Gynaecology and Obstetrics, Radboudumc, Geert Grooteplein Zuid 10, Nijmegen, GA 6525 The Netherlands; 2 Department of IQ Health, Radboudumc, Kapittelweg 54, Nijmegen, EP 6525 The Netherlands; 3 Department of Genetics, Radboudumc, Geert Grooteplein Zuid 10, Nijmegen, GA 6525 The Netherlands

**Keywords:** BRCA1, BRCA2, Carcinoma, Gynaecologic Surgical procedures, Ovarian Cancer, Prevention

## Abstract

Epithelial ovarian cancer (EOC) is the most lethal type of gynaecological cancer, due to lack of effective screening possibilities and because the disease tends to metastasize before onset of symptoms. Women with an increased inherited risk for EOC are advised to undergo a risk-reducing salpingo-oophorectomy (RRSO), which decreases their EOC risk by 96% when performed within guideline ages. However, it also induces premature menopause, which has harmful consequences. There is compelling evidence that the majority of EOCs originate in the fallopian tube. Therefore, a risk-reducing salpingectomy with delayed oophorectomy (RRS with DO) has gained interest as an alternative strategy. Previous studies have shown that this alternative strategy has a positive effect on menopause-related quality of life and sexual health when compared to the standard RRSO. It is hypothesized that the alternative strategy is non-inferior to the standard RRSO with respect to oncological safety (EOC incidence). Three prospective studies are currently including patients to compare the safety and/or quality of life of the two distinct strategies. In this article we discuss the background, opportunities, and challenges of the current and alternative strategy.

## Preventing epithelial ovarian cancer

The incidence of epithelial ovarian cancer (EOC) worldwide is 314,000 a year and the disease results in 207,000 deaths annually [1]. EOC has a 5-year overall survival rate of 50%, and ranges from 93% in women with localized disease to 31% in women with advanced stage disease [1]. The majority of patients present with an advanced stage disease, because women with early stage disease are often asymptomatic or have nonspecific symptoms [2]. This results in ovarian cancer being the most lethal type of gynaecological cancer [1]. Screening with ultrasound and/or the tumour marker Cancer Antigen-125 (CA125) have not been proven effective in reducing mortality both in high-risk women and the general population [3, 4].

EOC entails not only cancer in the ovaries, but is rather a collective term for carcinomas of the ovaries, fallopian tubes, and the peritoneum [5]. The most prevalent type of EOC is high grade serous carcinoma (HGSC), which accounts for 65–75% of all ovarian cancers. Other types are less prevalent and entail clear cell (12%), endometrioid (11%), low-grade serous (3%) and mucinous carcinomas (3%) [6]. HGSC is hypothesized to develop from tissue embryologically derived from the Müllerian ducts. These tissues include the uterus, fallopian tubes, and the upper part of the vagina [7, 8].

Germline genetic testing in women diagnosed with EOC demonstrated that ~ 13.5% carry a pathogenic variant (PV) that increases ovarian cancer risk; ~11% in *BRCA1/2* and ~ 2.5% in other moderate penetrant genes such as *RAD51C/D, BRIP1*, and *PALB2* [9]. Women with a PV in *BRCA1* and *BRCA2* have a lifetime risk of 35–45% and 10–20% for ovarian cancer, respectively. PVs in *RAD51C/D, BRIP1*, or *PALB2*, are associated with an EOC lifetime risk of 5–13%, ~ 6%, and 3–5%, respectively [9–13]. In PV carriers the proportion of EOC with a HGSC subtype is higher than in the general population, namely 90% in *BRCA1/2-*PV carriers compared to 65–75% in the general population [6, 14]. In addition to EOC, *BRCA1*- and *BRCA2-*PV carriers have a 55–72% and 45–69% breast cancer risk. The lifetime breast cancer risks for PV carriers in *RAD51C/D* or *PALB2* are estimated to be 14–29% and 44–63%, respectively [15, 16]. *BRIP1* is not associated with a higher lifetime risk of breast cancer [17].

Since current screening options are proven not to be effective in reducing EOC mortality, focus has shifted towards prevention [4]. International guidelines recommend surgical removal of both ovaries and fallopian tubes in moderate- and high-risk women: a risk-reducing salpingo-oophorectomy (RRSO). The estimated lifetime risk for EOC determines eligibility for and timing of risk-reducing surgery, since it s recommended to perform the surgery before the incidence of EOC starts to rise. This is at age 35–40 *(BRCA1)*, 40–45 (*BRCA2*) or 45–50 (*RAD51C/D, BRIP1)* [18]. For *PALB2-*PV carriers, risk reducing surgery can be considered after an individual risk assessment incorporating family history and age [19]. RRSO is proven effective in reducing ovarian cancer risk with 80–96%, expected to be most effective when performed before exceeding the maximum guideline age [20, 21].

However, RRSO also has disadvantages, the main one being the induction of acute surgical menopause when performed at premenopausal age [22]. Premature menopause has side effects in both the short and long term, which impair quality of life [23]. In the short-term side-effects consist of hot flashes, night sweats, sleeping difficulties, loss of sexual desire, and vaginal dryness. Long term effects are an increased risk of osteoporosis, cardiovascular disease, and neurocognitive problems such as Parkinsonism or Parkinson’s disease [24–27]. The Harmony study is an ongoing trial, aiming to assess the long-term cardiovascular, cognitive, urologic, and sexual effects of premenopausal RRSO in a multi-centre and cross-sectional study [28]. In this study, there was no notable difference between the pre- and postmenopausal RRSO group regarding cognition 18 years post-surgery [29]. However, women with a pre-menopausal RRSO had a slightly higher chance of urinary incontinence and experienced more sexual discomfort and vaginal dryness than the postmenopausal group [30, 31].

Hormonal replacement therapy (HRT) is known to mitigate menopausal symptoms. It is recommended to use HRT after RRSO until the age of natural menopause, provided that patients do not have a contra-indication, such as breast cancer in their medical history [32, 33]. Nebgen et al. showed the importance of women being guided, and state that clinical care should focus on safe options for symptom management and optimisation of long-term health [34]. Worldwide, the uptake of RRSO varies from 17 to 98.5%. A systematic review by Park et al. identified several factors which were associated with the decision to undergo RRSO, such as age, *BRCA1/*2-PV carrier status, perceived risk/worry/anxiety for ovarian cancer and perceived advantages of RRSO. It is assumed that socio-cultural variations in which patient preferences, physician preferences, healthcare systems and access to care differ, have an impact on this variation in uptake [35–39]. Additionally, stopping of ovarian cancer screening can have impact on RRSO uptake. In a study by Van Driel et al., the percentage of women who underwent RRSO within the recommended age range increased when ovarian cancer screening was no longer offered [40].

## Serous tubal intraepithelial carcinoma: a HGSC precursor lesion

**Fig. 1:**
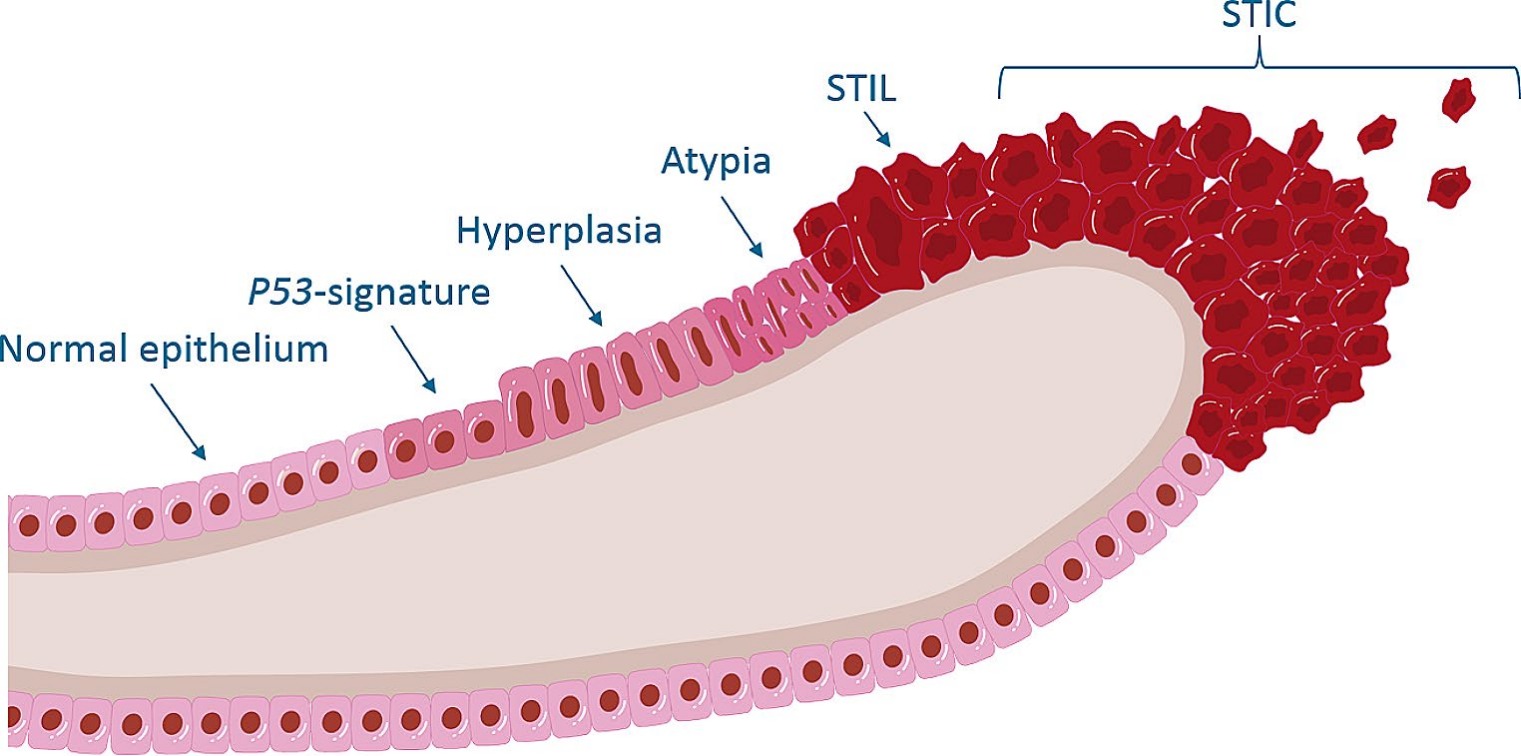
Spectrum of fallopian tube abnormalities

Precursor lesions of HGSC are found in the fallopian tube which are called Serous Tubal Intraepithelial Carcinomas (STIC) [41]. A STIC has the same morphological characteristics as HGSC, except for the stromal invasion which is absent in STIC. It is characterised by aberrant immunohistochemical staining for P53 and Ki-67, but this is not required for diagnosis [42]. Most STICs are found at the distal or fimbriated end of the fallopian tubes. The spectrum of fallopian tube abnormalities in the fimbriated end from least to most morphological changes consists of normal epithelium, P53-signature, hyperplasia, atypia, serous tubal intraepithelial lesion (STIL), STIC (Fig. 1) and lastly an invasive carcinoma. So far, no precursor lesions of ovarian cancer have been found in the ovaries [43].

STICs can be detected together with HGSC or without concurrent malignancy in isolated form. Detection of STIC and HGSC together is observed in 11–61% of HGSC’s [44]. An isolated STIC is detected in about 3–4% of high-risk women undergoing RRSO and in < 0.01% of the general population [42, 45]. Current practice when diagnosing an isolated STIC after salpingectomy is removal of the ovaries [46]. However, there are no guidelines for management after isolated STIC diagnosis after RRSO [47], partly due to the low prevalence which hinders research with a sufficient sample size [48].

Diagnosing a STIC has been proven difficult for several reasons: the small size of a STIC, its low prevalence, and the absence of standardized diagnostic criteria [48]. Aspects that could contribute to the low prevalence are: (1) underdiagnosing STIC because of sampling error due to the small size of the lesions, (2) overgrowth of cancer in the fallopian tubes in advanced stage EOC, (3) reporting bias, since there are no clinical consequences when a STIC is detected simultaneously with HGSC, (4) some early serous proliferations may skip the stage of STIC and function as a direct precursor of HGSC, the ‘precursor escape model’ [49].

The risk of developing a peritoneal carcinomatosis after detection of STIC at RRSO is strongly increased compared to women without a detected STIC, respectively 27.5% and 0.9% [50]. This is disconcerting, given a STIC is considered a non-invasive lesion. The prognosis of peritoneal carcinomatosis is poor, comparable to the 5-year survival in patients with stage III/IV EOC, which is 50% [1]. This underlines the importance of improving our understanding of the etiology and consequences of STIC.

## Risk-reducing salpingectomy with delayed oophorectomy

Knowledge on the role of the fallopian tubes in the development of HGSC improved since 2001. The progressing knowledge combined with the harmful consequences of premature menopause after RRSO shifted attention to a potential alternative strategy for the prevention of EOC: a risk-reducing salpingectomy (RRS) with a delayed oophorectomy (DO). It is hypothesized that RRS with DO is non-inferior to RRSO when considering EOC risk [51].

This alternative strategy has several potential advantages: (1) delaying surgical premature menopause and (2) EOC risk reduction may start earlier compared to the standard RRSO. This is because women may have their RRS at a relatively younger age compared to RRSO. Since the only function of the fallopian tube is to provide a connection between ovary and uterus to facilitate conception, natural conception is no longer possible after RRS [52]. This allows women to have their RRS as soon as childbearing is complete [46, 53]. This is often before the lower limit of the RRSO guideline age. A disadvantage of this alternative strategy is that women must undergo two separate surgeries with corresponding (limited) surgical risks.

In 2015, the Dutch ‘Early TUbectomy with delayed oophorectomy to improve quality of life as alternative for risk-reducing salpingo-oophorectomy in *BRCA1/2* PV carriers’ (TUBA) study started. This study investigates menopause-related quality of life (QoL) in women who underwent RRS with DO and compared this to women who underwent RRSO. One year after surgery, women who underwent RRS reported a better menopause related QoL compared to the RRSO group (with and without HRT) [23]. The WISP study started in 2016 and investigates sexual functioning in women who underwent RRS with DO compared to women who underwent RRSO [54].

A review by Perez et al. on patients’ perspectives showed that among *BRCA1/2*-PV carriers there is a high level of acceptance for RRS [55]. Avoidance of surgical menopause, preservation of fertility, concerns about sexual dysfunction, a family history of breast cancer, and the avoidance of HRT were facilitating factors in the acceptance. Barriers were the unknown effect on oncological safety, surgical timing, and surgical complications. Ghezelayagh et al. showed that *BRCA1/2*-PV carriers are satisfied with their decision after undergoing RRS. After surgery, women perceived their lifetime EOC risk as having decreased by half [56].

Not only RRS with DO is investigated. Leblanc et al. (2011) were the first to evaluate a radical fimbriectomy with DO to prevent EOC. The origin of the hypothesized efficacy of the radical fimbriectomy lies in the finding that most abnormalities are found distally in the fallopian tubes. The pilot study showed promising results regarding incidence of EOC. However, the sample size is yet too small and duration of the follow-up too short to draw conclusions on safety [57, 58].

Currently, there are three prospective studies recruiting participants to investigate the safety and/or quality of life of RRS with DO in women with an increased risk for ovarian cancer. The first is the PROTECTOR trial which started in 2018 in centres throughout the United Kingdom [53]. The second study is the SoROCK trial, which started in 2020 in the United States [59]. The third trial is the TUBA-WISP II study, which started in 2020 [46]. This latter study is an international collaboration between the Dutch TUBA study group and the WISP study group from the United States. There are several differences between the three studies, such as number of arms, eligibility of types of PV carriers, and focus of the primary outcome (Table 1). Especially the upper age limit for undergoing RRS, which is a pivotal distinguishing factor among the three studies. Considering the development of EOC and the role of STIC in this oncogenic process, it is presumable that RRS at a younger age is more effective then at an older age. This could impact the outcome of the strategy’s safety in these trials.

**Table 1:** Characteristics of ongoing studies regarding risk-reducing salpingectomy with delayed oophorectomy as an alternative for RRSO

	PROTECTOR (ISRCTN25173360) [47]	SoROCK (NCT04251052) [53]	TUBA-WISP II (NCT04294927) [40]
Design	Prospective and preferential	Prospective and preferential	Prospective and preferential
Centres (n)	42	415	46
Countries (n)	1	2	12
Arms	1. RRSO 2. RRS with DO 3. No surgery (Screening)	1. RRSO 2. RRS with DO	1. RRSO 2. RRS with DO
Inclusion criteria	1. *BRCA1/2-, RAD51C/D-, BRIP1-* or *PALB2-*PV carrier or a strong family history*	1. *BRCA1-*PV carrier	1. BRCA1/2-, RAD51C/D-**, BRIP1** or *PALB2*-PV** carrier
	2. Premenopausal	2. Premenopausal or menopausal	2. Premenopausal
	3. Presence of at least one fallopian tube	3. Presence of at least once fallopian tube	3. Presence of at least one fallopian tube
	4. Age > 30	4. Age: 35–50	4. Age: 25–40 (*BRCA1*), 25–45 (*BRCA2*), 25–50 *(RAD51C/D, BRIP1*)
	5. Completed childbearing	5. Completion of childbearing through natural conception	5. Completed childbearing
Primary outcome	Sexual functioning	Time to development of incident HGSC, specifically ovarian, primary peritoneal or fallopian tube cancers	Cumulative incidence of ovarian cancer at target age 46 (*BRCA1*) and 51 (*BRCA2*)

* A family history with > 3 ovarian cancers on the same side of the family (i.e., maternal or paternal) in *BRCA*1/2 negative women OR > 2 ovarian cancers on the same side of the family in *BRCA*1/2 unknown women

** Not included in the power calculations for the primary outcome

A large number of participants and a long duration of follow-up are needed to accurately assess the safety of the alternative strategy. This is due to the relatively young age of participants, whilst EOC mostly occurs at an older age, and the low incidence of EOC after risk-reducing surgery.

Safety of this alternative strategy is based on the evidence that HGSC originates in the fallopian tubes [60]. However, there are several theories about how peritoneal cancer could occur after a salpingectomy where no pathological abnormalities are found. These include: (1) ovarian inclusion cysts, which contain tubal epithelium that could undergo the same morphological changes as the epithelium located in the fallopian tubes, and thus lead to ovarian cancer (2) the previously mentioned precursor escape model, combined with genomic catastrophe, which means a STIC suddenly develops with invasive and metastatic potential (3) sampling error or underdiagnosis of STIC. The validity and clinical relevance of these theories are currently unknown, but they might have implications for the safety of RRS with DO [49, 61, 62].

To determine the risk of ovarian cancer after RRS with DO, the cumulative ovarian cancer risk was calculated in an earlier statistical model. In this study a risk-reducing effect of 65% for RRS and 80–96% for RRO were assumed. This model demonstrated that postponing RRO by five years beyond current guideline ages (45 for *BRCA1*, 50 for *BRCA2)* results in a cumulative risk of ovarian cancer at age 70, that is comparable to the risk after RRSO at guideline age. In case these assumptions are incorrect and RRS has no effect on ovarian cancer incidence, ovarian cancer risk will increase with a maximum of 2.3% points for *BRCA1* and 1.2% point for *BRCA2-*PV carriers, respectively, depending on age at surgery [51]. This risk reduction estimate is based on studies reporting that 65% of ovarian cancers are of the HGSC subtype [6]. Since more recent studies showed that this percentage is up to 90% in *BRCA*1/2-PV carriers, the effect of RRS on ovarian cancer risk reduction might even be higher [14].

## Future perspectives

At this moment, RRS with DO is advised to only be performed within a clinical trial. This is to ensure that participants are properly monitored, since the safety of this alternative strategy has not been proven yet. Selection criteria of participants differ in studies that are currently recruiting participants which affect the strategy and outcome of the studies. To determine which women may benefit from the alternative strategy, a few elements must be considered. Prevention via early salpingectomy is hypothesized to be most effective in groups where HGSC is the dominant EOC subtype. In some patients the dominant EOC subtype is unknown, due to for example no known PV, or a rare PV. The dominant subtype can also be different than HGSC, for example in Lynch Syndrome, in which endometroid and clear cell subtype are most prevalent [63, 64]. This may lead to the strategy being less effective in preventing EOC in these women [65]. Furthermore, previous research has shown that RRSO at the age of forty is only cost-effective for pre-menopausal women from an estimated EOC lifetime risk of 4% [65]. In PVs that are associated with a risk below 4%, surgical risks could outweigh the benefits.

To estimate ovarian cancer risk, the usage of risk prediction tools has become more common. One of the most frequently used tools is CanRisk, which provides breast and ovarian cancer risks [66, 67]. It combines several risk factors, such as age, family history, and PVs of rare and common genes (using polygenic risk scores). The model provides a more personalised approach for cancer risk estimation, which may lead to more distinctive prevention strategies. Since the use of such tools is relatively new, the role of CanRisk in preventive care has not been clearly defined yet. More research into this subject is needed.

Because of the early age at which a salpingectomy can be performed, questions regarding women who have not completed childbearing, but would prefer risk-reducing surgery, have arisen. Natural conception is no longer possible after RRS, but pregnancy through in vitro fertilization (IVF) or Intra Cytoplasmic Sperm Injection (ICSI) is. In some countries preimplantation genetic testing is available for *BRCA1/2*-PV carriers [68, 69]. However, these methods have several disadvantages compared to natural conception. They do not come with a guaranteed assurance of success, which means women could end up with an unfulfilled child wish. Additionally, these methods have a higher risk for pregnancy complications, are expensive, and not always fully covered by insurances. Moreover, the process to get pregnant can be a burden for women. This is due to possible psychological stress as well as physical complaints [70–72]. From an ethical standpoint, the safety of RRS has yet to be proven, so it is still an ‘experimental’ surgery. To restrict women to fertility treatment with significant side effects because of still unproven surgery is questionable. After RRSO, IVF or ICSI are no longer possible. Social freezing could be an alternative option for this group. Women could harvest and freeze their oocytes or an embryo prior to risk-reducing surgery and use them afterwards. However, this also comes with disadvantages: additional costs, lower odds of conceiving, and the complex psychological and social impact of social freezing on patients [73, 74].

In current guidelines, HRT is advised after RRSO. However, there is still a lot unknown about HRT in general, such as the optimum regarding dose, route of administration, and duration of treatment [34]. For a long time, all types of previous breast cancer have been a strict contra-indication for usage of HRT. However, recent research has shown that the risk of breast cancer recurrence is significantly increased in women who have hormone receptor-positive breast cancer, but not in women who had hormone receptor-negative tumours [75, 76]. This has affected management of HRT after previous breast cancer and may have implications on policy in the future. It is crucial to carefully consider all pros and cons and engage in shared decision-making when considering HRT.

To further optimize preventive care for women with an increased inherited risk, it is of the utmost importance to face the challenges regarding diagnosis and follow-up of STIC [48]. More research is necessary to take the next step in understanding the pathophysiology, create standardized diagnostic criteria, and discover its long-term consequences. A major challenge is the scarcity of STIC, which is why international collaboration to obtain a well-structured data collection is necessary. An international STIC data registry is being established in the Netherlands (by Radboud University Medical Centre) in which participation is possible.

If RRS with DO is proven to be safe, it might be the first step to an even more patient friendly preventive strategy: a salpingectomy only, without an oophorectomy. Women would only have to undergo one surgery, whilst avoiding the detrimental consequences of premature menopause. Though, at this point, there is not enough evidence to support this approach. Possibly, when RRS with DO is proven to be safe, a study with a salpingectomy alone, without delayed oophorectomy will be the next step [77].

## Conclusions

The risk-reducing salpingectomy with delayed oophorectomy is a strategy that has high-potential as an alternative for RRSO. Quality of life appears to be better in the RRS with DO group compared to the standard RRSO. Ongoing studies are still assessing the safety of the alternative strategy in relation to EOC incidence. Data on safety is expected to be available in 2036.

## Data Availability

No datasets were generated or analysed during the current study.
